# The Circulating miRNA Profile of Chronic Hepatitis D and B Patients Is Comparable but Differs from That of Individuals with HBeAg-Negative HBV Infection

**DOI:** 10.3390/v15112257

**Published:** 2023-11-15

**Authors:** Daniela Cavallone, Eric David B. Ornos, Gabriele Ricco, Filippo Oliveri, Barbara Coco, Piero Colombatto, Laura De Rosa, Leslie Michelle M. Dalmacio, Ferruccio Bonino, Maurizia Rossana Brunetto

**Affiliations:** 1 Hepatology Unit and Laboratory of Molecular Genetics and Pathology of Hepatitis Viruses, Reference Centre of the Tuscany Region for Chronic Liver Disease and Cancer, University Hospital of Pisa, Via Paradisa 2, 56124 Pisa, Italy; danielacavallone@hotmail.com (D.C.); ebornos@up.edu.ph (E.D.B.O.); riccogabriele@gmail.com (G.R.); f.oliveri@ao-pisa.toscana.it (F.O.); b.coco@ao-pisa.toscana.it (B.C.); p.colombatto@ao-pisa.toscana.it (P.C.); ferruccio.bonino@unipi.it (F.B.); 2 Institute of Biostructure and Bioimaging, National Research Council, Via De Amicis 95, 80145 Naples, Italy; 3 Department of Medical Microbiology, College of Medicine, University of the Philippines Manila, Pedro Gil Street, Ermita, Manila 1000, Philippines; 4 Fondazione Italiana Fegato (FIF), 34149 Trieste, Italy; 5 Institute of Clinical Physiology, National Research Council, 56124 Pisa, Italy; laura.derosa.95@gmail.com; 6 Department of Information Engineering and Computer Science, University of Trento, 38123 Trento, Italy; 7 Department of Biochemistry and Molecular Biology, College of Medicine, University of the Philippines Manila, Pedro Gil Street, Ermita, Manila 1000, Philippines; lmdalmacio@up.edu.ph; 8 Department of Clinical and Experimental Medicine, University of Pisa, 56126 Pisa, Italy

**Keywords:** MiR-B-Index, Hepatitis D Virus, Hepatitis B Virus, HDV-RNA, HBV-DNA

## Abstract

miRNAs circulating in whole serum and HBsAg-particles are differentially expressed in chronic hepatitis B (CHB) and HBeAg-negative-HBV infection (ENI); their profiles are unknown in chronic hepatitis D (CHD). Serum- and HBsAg-associated miRNAs were analyzed in 75 subjects of 3 well-characterized groups (CHB 25, CHD 25, ENI 25) using next-generation sequencing (NGS). Overall miRNA profiles were consonant in serum and HBsAg-particles but significantly different according to the presence of hepatitis independently of Hepatitis D Virus (HDV)-co-infection. Stringent (Bonferroni Correction < 0.001) differential expression analysis showed 39 miRNAs upregulated in CHB vs. ENI and 31 of them also in CHD vs. ENI. miRNA profiles were coincident in CHB and CHD with only miR-200a-3p upregulated in CHB. Three miRNAs (miR-625-3p, miR-142-5p, and miR-223-3p) involved in immune response were upregulated in ENI. All 3 hepatocellular miRNAs of MiR-B-Index (miR-122-5p, miR-99a-5p, miR-192-5p) were overexpressed in both CHB and CHD patients. In conclusion, CHD and CHB patients showed highly similar serum miRNA profiling that was significantly different from that of individuals with HBeAg-negative infection and without liver disease.

## 1. Introduction

Hepatitis D virus (HDV), the aetiologic agent of hepatitis D, is the progenitor of Kolmioviridae, the only family of a newly proposed realm named Ribozyviria [1,2,3,4]. HDV has unique biological properties: its virion consists of a 36–39 nm particle without nucleocapsid structure, coated by the surface antigen of Hepatitis B Virus (HBsAg) [1,2,3,4,5]. Such a structure allows HDV to enter into the hepatocytes using the Hepatitis B Virus (HBV) receptor, the sodium taurocholate co-transporting polypeptide (NTCP). The HDV genome is a circular, single-stranded RNA with negative polarity of about 1700 nucleotides in length [5]. Within the infected cell nucleus, genomic HDV-RNA replicates via a rolling circle mechanism that is common to circular RNA and plant viroids [6,7,8,9]. It generates an antigenomic multimer that is self-cleaved by the intrinsic ribozyme properties of HDV-RNA [10,11] and borrows nuclear RNA polymerase II for transcription [12]. Eight different HDV genotypes have been identified so far, with up to 35% divergence and 81–89% homology in nucleotide sequences within the same genotype [13]. Since early studies, it has become evident that hepatitis D occurs only in HBV carriers, and the outcome of liver disease is influenced by the phase of chronic HBV infection [14,15]. In patients with chronic hepatitis B, HDV accelerates the course of liver disease toward cirrhosis, particularly in hepatitis B “e” antigen (HBeAg)-positive patients [14,15,16,17]. Several studies indicate that the interplay between HBV and HDV is complex, suggesting that the replication of HBV and its transcriptional activity play a major role in fostering HDV pathogenicity [16,18,19,20].

Upon replication mediated by human RNA polymerase II, HDV-RNA is released from infected hepatocytes as ribonucleoprotein complex (RNP) together with delta antigen (HDAg) assembled within the HBsAg envelope. In a similar fashion, HBsAg particles carry hepatocellular microRNAs (miRNAs) generated by the same RNA polymerase II and export them in RNPs in addition to cell-derived particulate forms, exosomes, and micro-vesicles [21]. Consequently, the amount of circulating hepatocellular miRNAs is significantly higher in HBsAg carriers than in normal individuals or patients with chronic hepatitis C [22]. Thus, circulating miRNA profiles of HBsAg carriers are specifically enriched by hepatocellular miRNA, and their study provides an indirect opportunity to characterize and identify instant post-transcriptional regulatory networks associated with specific pathophysiologic conditions of the liver. Accordingly, circulating miRNAs were shown to be differentially expressed between HBeAg-negative CHB patients and HBV carriers with HBeAg-negative infection, and a specific hepatocellular miRNA signature was associated with both naturally-occurring and therapy-induced immune control of HBV infection, distinguishing carriers of the HBeAg-negative infection from CHB patients [23]. The present study aimed to characterize and compare the circulating miRNA profiles of CHD patients to those of patients with HBeAg-negative CHB and individuals with HBeAg-negative infection.

## 2. Materials and Methods

### 2.1. Subjects 

Serum samples were obtained from 75 (37 males, median age 44.0 years, 17.9/70.1 and 38 females, median age 46.4 years, 23.9/70.6) well-characterized HBsAg carriers and were followed up at the Hepatology Unit of the University Hospital of Pisa. All the patients were HBeAg-negative/Anti-HBe-positive; 96.8% were infected with HBV genotype D, and all were without histological and/or imaging signs of cirrhosis.

The study was approved by the local Ethics Committee of the University Hospital of Pisa (CEAVNO—n° 22971) and conducted according to the principles expressed in both the Declarations of Helsinki and Istanbul. All patients provided informed written consent. HBsAg carriers were classified according to the biochemical and viral profiles. In case of low HBV-DNA levels (<20,000 IU/mL) and normal transaminases (ALT) at the 1^st^ observation, the HBsAg carriers were monitored for at least 1 year with three monthly blood tests for classification [24,25,26].

The study population included: (a) 25 individuals with HBeAg-negative/anti-HBe positive HBV infection (ENI; serum HBV-DNA persistently <2000 IU/mL, normal ALT and liver stiffness < 5 kPa by vibration controlled transient elastography (FibroScan^®^, Echosens, Paris, France)); (b) 25 HBeAg-negative/anti-HBe-positive chronic hepatitis B (CHB) patients (HBV-DNA > 2000 IU/mL, persistently or intermittently elevated ALT but without signs of cirrhosis at histology and/or ultrasound and liver stiffness < 12 kPa); (c) 25 HBeAg-negative/anti-HBe-positive/anti-HDV-positive/HDV-RNA-positive chronic hepatitis D (CHD) patients without signs of cirrhosis at histology and/or ultrasound and liver stiffness < 12 kPa. Their demographic and clinic pathologic features are reported in Table 1.

### 2.2. Assays

Serum ALT levels were tested using routine biochemistry at the Central Laboratory of the Hospital using a Cobas Analyser, Roche. HBsAg, anti-HBs, anti-HBc, IgM anti-HBc, HBeAg, anti-HBe, anti-HCV, anti-HDV, and anti-HIV were detected using commercially available immunoassays (Architect, Abbott laboratories, N. Chicago, IL, USA).

Serum HBsAg was quantified using the Architect HBsAg assay (Abbott Laboratories, dynamic range 0.05–250.0 IU/mL) after appropriate dilution when required. Serum HBV-DNA was quantified using the HBV-monitor-2.0 test on Cobas Amplicor until 2005 and on Cobas TaqMan thereafter (Roche Diagnostic Systems Inc., Mannheim, Germany). HBV genotype was characterized by direct sequencing of the small HBs region. Briefly, after HBV DNA extraction using Qiagen QIAamp DNA Mini Kit (Qiagen-Inc., Hilden, Germany), 5 µL of eluted DNA was amplified by PCR in 50 µL reaction volume. For the amplification two overlapping PCRs were performed using the following primers: P1-5′-TCACCATATTCTTGGGAACAAGA-3′/S1-2-5′-CGAACCACTGAACAAATGGC-3′ for the Pre-S region and LamEs-5′-GGATGTGTCTGCGGCGTTT-3′/Pol4as-5′-GGCATTAACGCAGGATAWCCACATTG-3′ for the small S. An internal primer was used for emi-nested PCR when needed: PS4-5′-GGAACAAGAGCTACAGCTTG-3′ and Pol181as-5′-GACCCACAATTCKTTGACATACTTTCC-3′ for the Pre-S and S regions, respectively. Thermal profile amplification was: pre-heating 15 min at 94 °C, 35 cycles including denaturation 20 s at 94 °C, annealing 20 s at 60 °C, and extension 30 s at 72 °C followed by 5 min at 72 °C. PCR products were then purified using an ExoSAp Purification System and directly sequenced through the chain termination method using the BigDye Terminator v1.1 Cycle Sequencing Kit (Applied Biosystem, Waltham, MA, USA). Sequences were analyzed and corrected using Chromas Lite software, version 2.6.6 (www.technelysium.com.au, accessed on 25 February 2022). Multiple alignments were performed using NPS @ Multalin software version 5.4.1 (https://npsa-pbil.ibcp.fr). Alignments and trees were generated, along with references to small HBsAg sequences of the 8 HBV genotypes (A–H) obtained from GenBank, with the MEGA 3.1 software package available at http://www.megasoftware.net/.

### 2.3. Isolation of Circulating HBsAg Particles

Circulating HBsAg particles were obtained via immunoprecipitation of 20 sera obtained form 5 ENI, 5 CHB, 5 CHD, and 5 controls without HBV infection and liver disease using anti-HBsAg-IgG conjugated with magnetic beads, as previously reported [23]. Briefly, 200 µL serum after appropriate dilution to 1000 IU/mL of HBsAg final, was incubated for 60 min at 37 °C with 67 µL of anti-HBsAg-IgG conjugated with beads (Dia.Pro). At the end of the incubation, three washes were carried out with 10 mM Tris-HCl buffer containing 0.1% BSA, 0.15 M NaCl, 1 mM EDTA, 0.1% NaN3, and 0.025% Proclin 300 pH 7.5.Finally,the HBsAg particles were resuspended in 200 µL PBS buffer.

### 2.4. miRNA NGS Reads Bioinformatical Analysis

Next-generation sequencing (NGS) was performed according to QIAGEN miRNA Sequencing Genomic Services protocols. Total RNA was extracted from 200 µL serum or 200 µL of HBsAg immunoprecipitated particles (HBsAg-IP) using an miRNeasy-kit (Qiagen-Inc., Hilden, Germany). The library preparation was performed using the QIAseq miRNA Library Kit. Briefly, to prepare miRNA NGS libraries, 5 µL of total RNA was ligated with specifically designed adapters and then reverse transcribed to cDNA using a reverse transcription (RT) primer with unique molecular identifiers (UMI). Library amplification was performed via PCR (16 cycles) using a universal forward primer and indexing reverse primers. Then, the miRNA library was purified using a magnetic-bead-based method and quality controlled using capillary electrophoresis. Based on the quality of the inserts and the concentration measurements the libraries were pooled in equimolar ratios. The library pool(s) were quantified using qPCR and then sequenced on NextSeq instrument (Illumina Inc., San Diego, CA, USA) according to the manufacturer’s instructions (1 × 75, 1 × 8). Raw data were demultiplexed and FASTQ files for each sample were generated using the bcl2fastq software version 2.20 (Illumina Inc., San Diego, CA, USA).

### 2.5. Read Mapping and Quantification of Gene Expression

All primary analysis was carried out using CLC Genomics Server 21.0.4. The workflow “QIAseq miRNA Quantification” of CLC Genomics Server with standard parameters was used to map the reads to miRBase version 22. Briefly, the reads were processed by (1) trimming the common sequence, UMI, and adapters and (2) filtering reads with lengths < 15 nt or >55 nt. They were then deduplicated using their UMI. Reads were grouped into UMI groups when they (1) started at the same position based on the end of the read to which the UMI was ligated (i.e., Read2 for paired data), (2) were from the same strand, and (3) had identical UMIs. Groups that contained only one read (singletons) were merged into non-singleton groups if the singleton’s UMI could be converted to a UMI of a non-singleton group by introducing an SNP (the biggest group was chosen). All reads that did not map to miRBase, neither with perfect matches nor as isomiRs (maximum 2 mismatches and/or alternative start/end position of 2 nt), were mapped to the human genome GRCh38 with ENSEMBL GRCh38 version 97 annotation. This was carried out using the “RNA-Seq Analysis” workflow of CLC Genomics Server with standard parameters. The “Empirical analysis of DGE” algorithm of the CLC Genomics Workbench 21.0.4 was used for differential expression analysis with default settings. It is an implementation of the “Exact Test” for two-group comparisons developed by Robinson and Smyth [27] and incorporated in the EdgeR Bioconductor package [28]. For all unsupervised analysis, only miRNAs were considered, with at least 10 counts summed over all samples. A variance stabilizing transformation (VST) was performed on the raw count matrix using the R package DESeq2 version 1.28.1, and 500 genes with the highest variance were used for the principal component analysis. The variance was calculated agnostically to the pre-defined groups (blind = TRUE), and 35 genes with the highest variance across samples were selected for hierarchical clustering.

### 2.6. MiR-B-Index

A miRNA calculator (MiR-B-Index) was previously developed by using single RT-q-PCR for the 3 hepatocellular miRNAs (miR-122-5p, miR-99a-5p, miR-192-5p) with the most significant differential expression in ENI versus CHB and 3 additional miRNAs (miR-335-5p, miR-126-3p, and miR-320a) as endogenous controls to account for RNA input variation and/or other technical variations within the profiling platform [23]. In the current study for the MiR-B-Index calculation, we used CPM (count per million mapped reads) obtained from NGS instead of Cq values (obtained by RT-q-PCR).

## 3. Results

### 3.1. NGS and Quality Controls

For each sample, on average, ~17 million reads were sequenced, and the produced data showed an excellent sequencing quality, with a Phred score > 30. The distribution of read length had prominent peaks at ~21 nt and ~32 nt, suggesting that a large proportion of the sequencing data was represented by miRNA. Following unique molecular identifier (UMI) deduplication, an average UMI group size between 1.53–2.61 was observed across all samples; the deduplicated reads were mapped to miRBase. The percentage of reads that were successfully mapped ranged from 1.22–62.36% which is the level of mapping typically given by good-quality serum specimens. All reads that did not map to miRBase were mapped to the human genome. The mapping rate was between 64.94–78.0%, with the largest proportion of these reads mapping to protein-coding genes.

### 3.2. miRNA Profiling of HBsAg Particles

**Figure 1:**
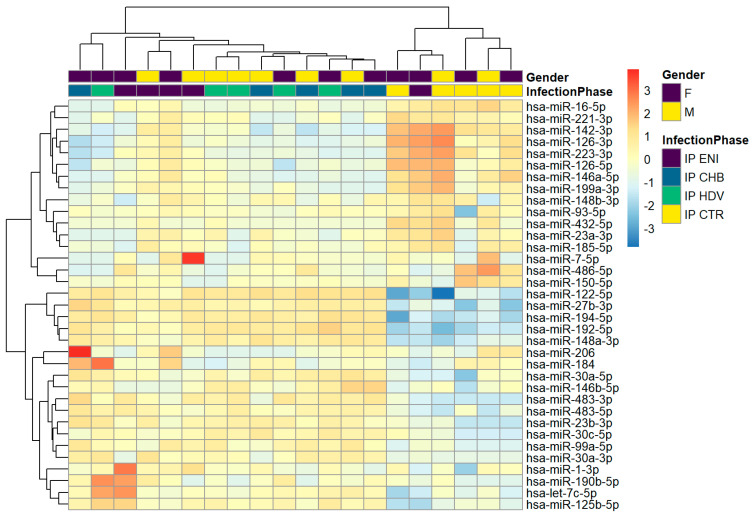
Hierarchical clustering in HBsAg-immunoprecipitated particles. A variance-stabilized transformation was performed on the raw count matrix, and 35 genes with the highest variance across samples were selected for hierarchical clustering. Each row represents one gene, and each column represents one sample. The color represents the difference of the count value to the row mean.

The analysis of hierarchical clustering of the 20 immunoprecipitated samples showed that miRNA profiling of CHB clusters with that of CHD, whereas ENI showed a separate profile more similar to control (HBsAg negative) samples without hepatitis infection (Figure 1). Thirty-five miRNAs (miR-16-5p, miR-221-3p, miR-142-3p, miR-126-3p, miR-223-3p, miR-126-5p, miR-146a-5p, miR-199a-3p, miR-148b-3p, miR-93-5p, miR-432-5p, miR-23a-3p, miR-185-5p, miR-7-5p, miR-486-5p, miR-150-5p, miR-122-5p, miR-27b-3p, miR-194-5p, miR-192-5p, miR-148a-3p, miR-206, miR-184, miR-30a-5p, miR-146b-5p, miR-483-3p, miR-483-5p, miR-23b-3p,miR-30c-5p, miR-99a-5p, miR-30a-3p, miR-1-3p, miR-190b-5p, let-7c-5p, miR-125b-5p) showed a higher variability among the different groups. The 3 liver-related miRNAs (miR-122-5p, miR-99a-5p, and miR-192-5p) were upregulated in CHB and CHD samples but downregulated in ENI.

### 3.3. miRNA Profiling of Whole Serum

**Figure 2:**
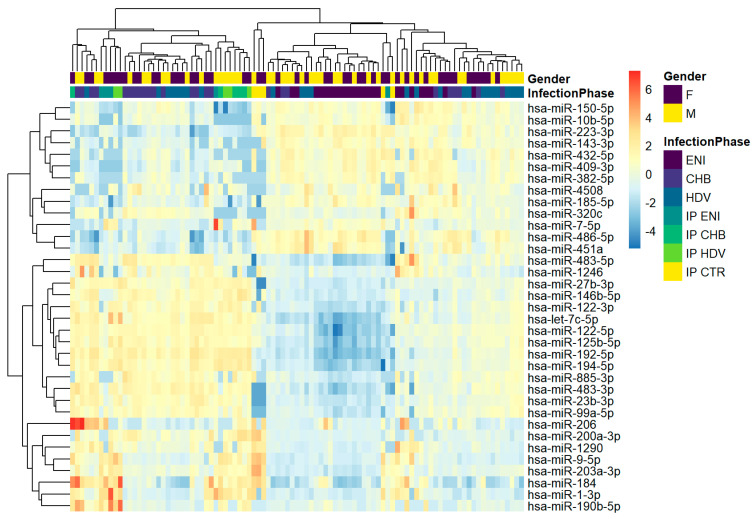
Hierarchical clustering of whole cohort study. The heatmap shows the result of the two-way hierarchical clustering of microRNAs and samples. Each row represents one gene, and each column represents one sample. The color represents the difference of the count value to the row mean.

The overall differential expression of the miRNAs resulting from the hierarchical clustering analysis performed on the 75 serum samples is reported in Figure 2. As for miRNA profiling obtained from HBsAg immunoprecipitated particles, the 3 liver related miRNAs were upregulated in CHB and CHD samples and downregulated in ENI.

### 3.4. Differential miRNA Expression among Chronic Hepatitis D and B Patients and Individuals with HBeAg Negative Infection

The total RNA counts successfully aligned in miRBase per subject groups were 1–3 log higher in serum specimens as compared to HBsAg pellets, and the miRNAs that showed the most variable expression among the three groups in the pellets were all present in the whole serum specimens. Thus, for the overall analysis of differential miRNA expression among the three groups, the results obtained from sera were used.

To plot the principal component analysis (PCA), a variance stabilizing transformation was performed on the raw count matrix, and 500 genes with the highest variance were used. The variance was calculated agnostically to the pre-defined groups. For each differential gene expression analysis, the result of the statistical test for each gene’s fold-change was plotted against its mean expression among all samples, and changes were defined and considered as significant for a Bonferroni test <0.001. Comparing the overall miRNA expression between males and females, only one miRNA, miR-7-5p, resulted differentially expressed and upregulated in males with levels of significance (Bonferroni test, 0.006) barely outside the cutoff of significance. Thus, all the following analyses were controlled by gender.

**Figure 3:**
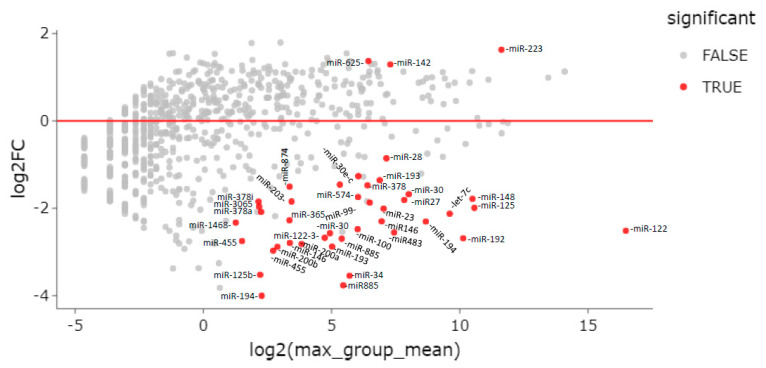
MA plot of differentially expressed genes (DEG). Differential expression of serum miRNAs between HBsAg carriers with HBeAg-negative infection (ENI) and CHB patients.

In total, 42 miRNAs were differentially expressed between ENI vs. CHB patients—39 downregulated and 3 upregulated (miR-625-3p, miR-142-5p, and miR-223-3p) in ENI (Table 2, Figure 3). 

**Figure 4:**
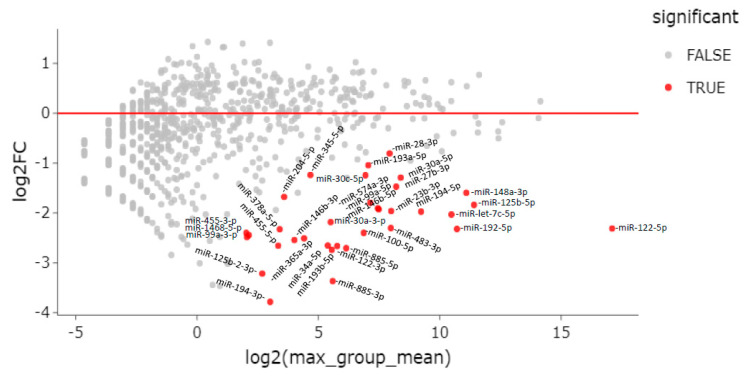
MA plot of differentially expressed genes (DEG). Differential expression of serum miRNAs between HBsAg carriers with HBeAg-negative infection (ENI) vs. CHD patients.

When comparing ENI to CHD patients, there were 34 differentially expressed miRNAs; all of them were downregulated in ENI (Table 3 and Figure 4).

**Figure 5:**
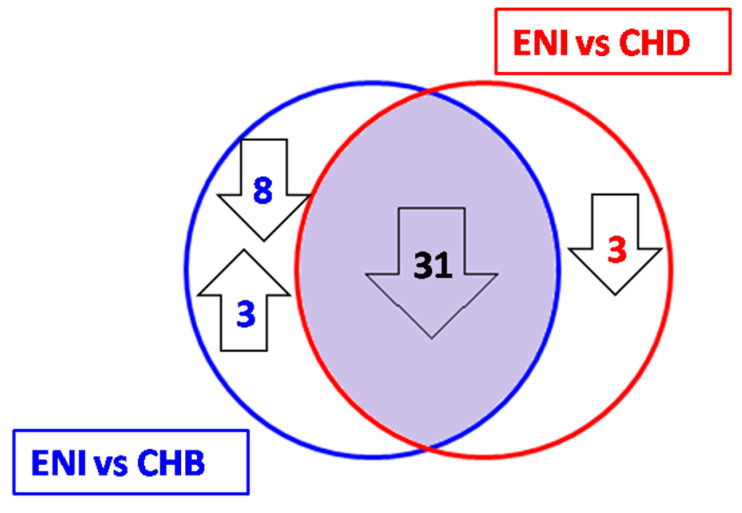
miRNAs were differentially expressed in individuals with HBeAg-negative infection (ENI), chronic hepatitis B (CHB), and chronic hepatitis D (CHD) patients; 31 miRNAs were downregulated in ENI when compared to both CHB and CHD. An additional 8 miRNAs were downregulated and 3 were upregulated when comparing ENI to CHB, and 3 were downregulated in the comparison between ENI and CHD.

Interestingly, 31 of 34 miRNAs were also found to be downregulated when comparing ENI with CHB patients, and these represented 73.8% of the circulating miRNAs with statistically significant differential expression (Bonferroni test level < 0.001). The remaining 3 miRNAs (miR-204-5p, miR-345-5p, and miR-99a-3p) were also downregulated when comparing ENI with CHB, however without achieving statistical significance (Table 4 and Figure 5).

Eight of the 39 miRNAs that were downregulated in ENI vs. CHB did not show significant differential expression when comparing ENI with CHD patients (Table 5 and Figure 5).

Interestingly, the three miRNAs (miR-625-3p, miR-142-5p, and miR-223-3p) that were upregulated in ENI when compared to CHB showed the same trend toward upregulation in comparison with CHD but did not achieve statistical significance (Supplementary Table S1). Between the two groups of patients with CHB and CHD, only miR-200a-3p resulted highly differentially expressed and upregulated in CHB (Bonferroni test 9.20 × 10^−4^). The second- and third- most differentially expressed miRNAs were miR-206 and miR-1246, which were upregulated in CHB without achieving a statistical significance (Bonferroni test, 0.056 and 0.080).

In individuals with HBeAg-negative infection, miRNA expression was analysed according to serum HBV-DNA levels (<100 IU/mL vs. 100–2000 IU/mL) and only miR-206 (Bonferroni test, 3.31 × 10^−5^) was downregulated in ENI with lower viremia. The same miRNA was significantly downregulated in CHB and CHD patients with ALT levels below 100 U/L (Bonferroni test, 0.0014). The second- most differentially expressed miRNA according to ALT levels was miR-1246, but this was found at a much lower level of stringency (Bonferroni test, 0.011).

### 3.5. MiR-B-Index

**Figure 6:**
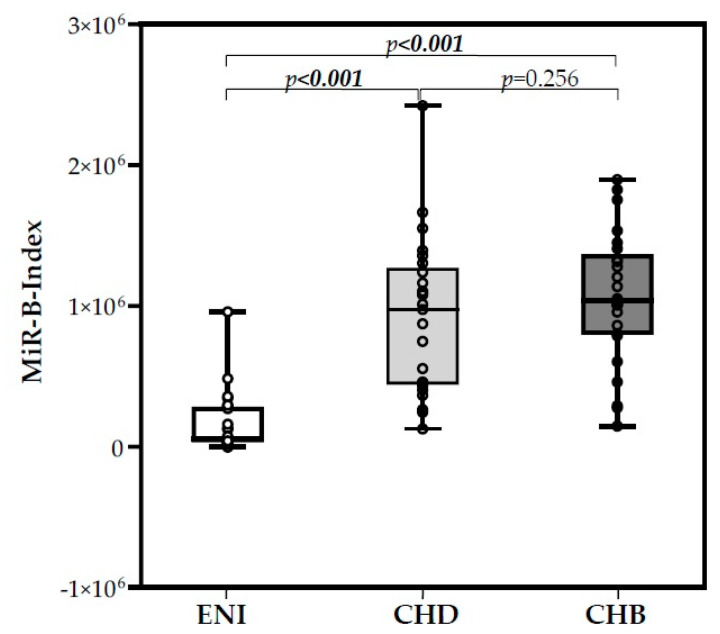
Box plot of the MiR-B-Index in the three groups of HBsAg-positive/HBeAg-negative individuals.

The expression levels of six miRNAs, three hepatocellular miRNAs (miR-122-5p, miR-99a-5p, miR-192-5p), and three control miRNAs (miR-126-3p, miR-335-5p, and miR-320a) were used to calculate the MiR-B-Index, as previously reported [23]. The mean MiR-B-Index value for ENI was 1.67 × 10^5^, that for CHB was 1.06 × 10^6^, and that for CHD was 9.10 × 10^5^ (Kruskal–Wallis, *p* < 0.001). MiR-B-Index values were comparable between CHB and CHD patients (Mann–Whitney, *p* = 0.256), whereas they were significantly different between CHB and ENI (Mann–Whitney, *p* < 0.001) and between CHD and ENI (Mann–Whitney, *p* < 0.001). The distribution of MiR-B-Index values in the three groups is reported in Figure 6.

## 4. Discussion

The analysis of the circulating miRNA profiles in 3 well-characterized groups of HBsAg positive/HBeAg-negative carriers showed significantly different patterns according to the presence or absence of liver disease independently of the co-infection with HDV (Figure 3 and Figure 4). Furthermore, as in previous reports, HBsAg-positive individuals showed significantly higher levels of miRNAs than controls (HBsAg-negative subjects), because of the miRNAs carried by HBsAg particles [21,22,23]. Nevertheless, the circulating miRNAs profiles in whole sera were consonant with those of immunoprecipitated HBsAg-particles, fostering the characterization of miRNA profile of the whole serum [22,23].

The stringent analysis of the differences between HBeAg-negative infection (ENI) and patients with chronic hepatitis B (CHB) revealed that 39 miRNAs were downregulated and 3 were upregulated in the former. Very interestingly, 31 of the 39 miRNAs downregulated between ENI and CHB were also downregulated at the same level of stringency in the comparison with CHD. Finally, the overall miRNA profiles of CHB and CHD patients were surprisingly coincident, as only one miRNA, miR-200a-3p, was found to be differentially expressed and upregulated in CHB patients.

The miRNA profile depicts the instant representation of the coincident expression of miRNAs that have reciprocal and interconnected actions influencing post-transcriptional gene expression and the epigenetic regulation of many differential pathways and feedback loops associated with an ongoing pathophysiologic process [29]. Particularly hepatic miRNA profiles had been shown to be involved in the regulation of liver metabolism, liver damage, progression of fibrosis, and oncogenesis [30]. Therefore, in HBsAg carriers, in which a specific enrichment in hepatocellular miRNAs is provided by circulating HBsAg particles, serum miRNAs may portray an indirect picture of the post-transcriptional epigenetic dysregulations linked with the ongoing infection and hepatocellular injury. As a consequence, the finding of consonant miRNA profiles in patients with CHB or CHD suggests that their virus-induced liver damage has similar pathogenetic pathways.

Actually, HDV replication is independent of HBV: HDV was shown to cause latent intrahepatic infection without liver damage following orthotopic liver transplantation [31,32,33,34]. More recently, both in vitro and in vivo studies demonstrated that HDV may persist during liver regeneration by transmission of HDV-RNA through cell division in absence of HBV [35,36]. Furthermore, in cell culture, the release and assembly of genomic HDV-RNA can be supported by the envelope proteins of vesicular stomatitis virus (VSV), hepatitis C virus (HCV), and Dengue virus [37,38]. However, so far, no clinical evidence supports the presence of HDV liver damage in absence of HBV infection [2]. Our findings support the hypothesis of shared pathogenic mechanisms of the underlying liver damage in both CHB and CHD patients.

Notwithstanding that to link differential expression of single serum miRNAs to specific pathophysiologic conditions is highly speculative and hazardous, it is interesting to note that the three miRNAs (miR-625-3p, miR-142-5p, and miR-223-3p) that were significantly upregulated in ENI vs. CHB appear to be involved in the regulation of immune response. Accordingly, it has been hypothesized that the secreted miRNAs could contribute to the crosstalk between hepatocytes and other cells [30]. Furthermore, a recent study demonstrated that miR-625-3p is upregulated in CD8+ T cells in vitro upon TCR-mediated activation, and its overexpression is tightly linked to the level of CD8+ T cell proliferation [39]. Furthermore, the high expression of miR-625-3p at an early time point during CD8+ T cell expansion after allogeneic stem cell transplantation suggested that miR-625-3p might be applicable as an intracellular biomarker for effective immune reconstitution [39]. miR-142-5p overexpression is linked with hyperimmune conditions [40], andmiR-223 was shown to play a central role in myeloid cells, especially neutrophil and macrophage differentiation, and in regulation of innate immunity [41]. These actions of the three miRNAs upregulated in HBeAg-negative infection are indeed consistent with the currently accepted theory that this phase of chronic HBV infection relies upon an effective immune control of HBV that instead is missing in CHB patients [23,42]. The three miRNAs (miR-345, miR-204, and miR-99a) significantly downregulated in ENI vs. CHD were also downregulated in ENI vs. CHB but at much lower levels of significance (namely Bonf.Cor. > 0.001 with *p*-values of 3.16 × 10^−03^, 2.84 × 10^−03^, and 1.37 × 10^−02^, respectively) (Table 4). Given the evidence that miR-204 is involved in the suppression of HBV replication, its highly significant upregulation in CHD raises the question of its possible contribution in the HDV induced inhibition of HBV replication [43,44].

The miRNA (miR-200a-3p) most differentially overexpressed in CHB vs. CHD patients was shown to be upregulated in HBV associated hepatocellular carcinoma (HCC) and negatively correlated with the expression of HBV-X oncogenic protein [45,46]. Interestingly, also the other two miRNAs (miR-206 and miR-1246) upregulated in CHB vs. CHD had been associated with HCC [47,48]. Age is usually a factor associated with an increased HCC risk [49], whether the older age of CHB patients (44.8 vs. 38.0 years, *p =* 0.017) may have a role in such a finding remains to be clarified; nevertheless, the high oncogenic potential of HBV infection is well known [50]. Finally, miR-206 was found to be downregulated in chronic hepatitis patients with ALT < 100 U/L, in accordance with previous findings in a different clinical setting [51]. Interestingly, miR-206 also showed a trend toward downregulation when comparing CHD vs. CHB patients; this could be explained by the lower ALT levels observed in the former (65 vs. 110 U/L, *p =* 0.001).

A highly significant overexpression of miR-122-5p, miR-99a-5p, and miR-192-5p was observed in the 50 patients with chronic hepatitis; the three miRNAs are the first-, second-, and sixth-most represented miRNAs of the human liver. miR-122 is considered to be a hepatocyte specific miRNA given that it represents more than 50% of hepatic miRNAs and that its expression in other tissues is negligible. Changes in its expression have been described in different pathological conditions: downregulation in HCC [52] and steatotic liver disease [53]. In the setting of viral infection, miR-122 appears to have opposite actions, as it promotes Hepatitis C Virus (HCV) replication by binding the region at the 5′-UTR of the HCV RNA genome, whereas it inhibits HBV replication through cyclin-G1-modulated p53 activity [54] and NDRG3 (a member of the N-myc downstream-regulated gene) [55] or directly by binding to HBV pregenomic RNA [56]. Accordingly, Wang et al. found that miR-122 levels are decreased in the liver of chronic HBV patients [54], oppositely to those observed in CHB sera samples [23,57,58,59]. This discrepancy highlights the complexity of the regulation of miR-122 liver expression and its release into circulation. Thus, further studies are requested to better address this issue. At variance for miR-99a-5p,positive regulation of HBV replication has been reported [60]. In addition, both miR-99a-5p and miR-192-5p have been identified as potential early biomarkers for hepatocellular carcinoma (HCC) [61,62]. Based on their overexpression, we previously included miR-122-5p, miR-99a-5p, and miR-192-5p in a score (MiR-B-Index) that we showed to be associated with both natural and therapy-induced immune control of HBV infection [23]. In the present study, we assessed the MiR-B-Index and found highly comparable values in CHB and CHD patients, whereas they were significantly different in individuals with HBeAg-negative infection without virus-induced liver disease (Figure 6). This finding further supports common pathogenetic mechanisms in liver injury of CHB and CHD patients.

The HBsAg positive carriers enrolled in the present study were well-characterized, pedigreed patients. However, the sample size was small. Therefore, further studies on larger cohorts of patients are required to confirm our findings and validate the use of MiR-B-Index in the management of HBsAg carriers.

## 5. Conclusions

In conclusion, the characterization of the differential profiles of circulating miRNAs reveals a highly similar pattern of miRNA expression (68.9% identical) in patients with chronic viral hepatitis B and D. On the contrary, the serum miRNA profile of carriers of HBeAg-negative infection differ significantly from both types of chronic viral hepatitis, showing a downregulation of 31 miRNA in ENI as compared to CHB and CHD and an upregulation of 3 miRNA involved in the modulation of immuneresponse.

## Figures and Tables

**Table 1:** Demographic, virological and biochemical characteristics according to patients’ classification.

		ENI (25)	CHB (25)	CHD (25)	*p*
Age	years	49.4 (28.5/70.1)	44.8 (31.8/70.6)	38.0 (17.9/60.4)	ENI vs. CHB: *p* = 0.184 ENI vs. CHD: ***p* < 0.003** CHB vs. CHD: ***p* = 0.017** Overall: ***p* = 0.004**
Gender	F M	13 (52.0) 12 (48.0)	12 (48.0) 13 (52.0)	13 (52.0) 12 (48.0)	ENI vs. CHB: *p* = 1.000 ENI vs. CHD: *p* = 1.000 CHB vs. CHD: *p* = 1.000 Overall: *p* = 0.948
Nationality	Italian Non-Italian	20 (80) 5 (20)	21 (84) 4 (16)	5 (20) 20 (80)	ENI vs. CHB: *p* = 1.000 ENI vs. CHD: ***p* < 0.001** CHB vs. CHD: ***p* < 0.001** Overall: ***p* < 0.001**
ALT (U/L)	Median (range)	21 (9/33)	110 (40/695)	65 (19/176)	ENI vs. CHB: ***p* < 0.001** ENI vs. CHD: ***p* < 0.001** CHB vs. CHD: ***p* = 0.001** Overall: ***p* < 0.001**
HBV Genotype	D Non-D unknown	25 (100) / /	25 (100) / /	10 (83.3) 2 (16.7) 13	ENI vs. CHB: **/** ENI vs. CHD: ***p* = 0.035** CHB vs. CHD: ***p* = 0.035** Overall: ***p* = 0.012**
HBsAg	Log IU/mL	2.71 (1.75/3.10)	3.75 (3.05/4.22)	3.89 (3.09/4.59)	ENI vs. CHB: ***p* < 0.001** ENI vs. CHD: ***p* < 0.001** CHB vs. CHD: *p* = 0.273 Overall: ***p* < 0.001**
HBV DNA	Log IU/mL	2.27 (0.70/3.26)	6.23 (4.61/8.04)	1.0 (0.70/7.61)	ENI vs. CHB: ***p* < 0.001** ENI vs. CHD: ***p* < 0.001** CHB vs. CHD: ***p* < 0.001** Overall: ***p* < 0.001**

Notes: Non-Italian natives were born: 13 (44.8%) in Albania; 3 (10.4%) in Moldova; 11 (37.9%) in Romania; and 2 (6.9%) in Ukraine. Significant (<0.05) *p* values in bold.

**Table 2:** Comparison of miRNA profiles in HBsAg carriers with HBeAg-negative infection (ENI) vs. CHB patients: a total of 42 miRNAs showed significant differential expression after Bonferroni correction (cut-off = 0.001).

Assay	Max Groupmean	Log2 Foldchange	Foldchange	*p*-Value	FDR *p*-Value	Bonferroni
hsa-miR-34a-5p	52.00	−3.54	−11.66	1.6 × 10^−30^	8.5 × 10^−28^	2.0 × 10^−27^
hsa-miR-885-3p	43.84	−3.76	−13.59	2.9 × 10^−30^	8.5 × 10^−28^	3.7 × 10^−27^
hsa-miR-192-5p	1121.40	−2.69	−6.44	8.2 × 10^−30^	1.6 × 10^−20^	1.0 × 10^−19^
hsa-miR-148-3p	1441.72	−1.78	−3.44	3.0 × 10^−21^	4.1 × 10^−19^	3.7 × 10^−18^
hsa-miR-27b-3p	227.80	−1.81	−3.51	3.5 × 10^−21^	4.1 × 10^−19^	4.5 × 10^−18^
hsa-miR-193b-5p	32.44	−2.88	−7.35	1.5 × 10^−20^	1.3 × 10^−18^	1.9 × 10^−17^
hsa-miR-194-5p	408.28	−2.30	−4.94	1.6 × 10^−20^	1.3 × 10^−18^	2.0 × 10^−17^
hsa-miR-885-5p	42.12	−2.70	−6.48	8.3 × 10^−19^	5.4 × 10^−17^	1.0 × 10^−15^
hsa-miR-100-5p	64.68	−2.48	−5.57	8.4 × 10^−19^	5.4 × 10^−17^	1.1 × 10^−15^
hsa-miR-30a-3p	30.60	−2.57	−5.94	2.3 × 10^−18^	1.3 × 10^−16^	2.9 × 10^−15^
hsa-miR-146b-5p	123.68	−2.30	−4.92	3.3 × 10^−18^	1.8 × 10^−16^	4.2 × 10^−15^
hsa-miR-122-5p	90,951.56	−2.51	−5.71	3.7 × 10^−18^	1.8 × 10^−16^	4.6 × 10^−15^
hsa-miR-30a-5p	257.48	−1.68	−3.20	7.8 × 10^−18^	3.5 × 10^−16^	9.8 × 10^−15^
hsa-miR-483-3p	173.36	−2.55	−5.87	4.8 × 10^−17^	2.0 × 10^−15^	6.0 × 10^−14^
hsa-miR-125b-5p	1516.76	−1.99	−3.97	4.6 × 10^−16^	1.8 × 10^−14^	5.8 × 10^−13^
hsa-miR-23b-3p	130.60	−2.01	−4.02	1.3 × 10^−14^	4.8 × 10^−13^	1.7 × 10^−11^
hsa-miR-146b-3p	10.36	−2.79	−6.93	3.0 × 10^−14^	1.0 × 10^−12^	3.7 × 10^−11^
hsa-miR-378a-3p	84.44	−1.48	−2.78	1.0 × 10^−13^	3.4 × 10^−12^	1.3 × 10^−10^
hsa-let-7c-5p	778.48	−2.12	−4.36	2.4 × 10^−13^	7.2 × 10^−12^	3.0 × 10^−10^
hsa-miR-193a-5p	117.48	−1.36	−2.56	2.6 × 10^−13^	7.4 × 10^−12^	3.2 × 10^−10^
hsa-miR-194-3p	4.84	−4.00	−16.02	5.7 × 10^−13^	1.6 × 10^−11^	7.2 × 10^−10^
hsa-miR-125b-2-3p	4.64	−3.52	−11.49	6.0 × 10^−13^	1.6 × 10^−11^	7.6 × 10^−10^
hsa-miR-200a-3p	14.20	−2.82	−7.05	1.2 × 10^−12^	3.1 × 10^−11^	1.6 × 10^−9^
hsa-miR-99a-5p	89.64	−1.87	−3.65	1.4 × 10^−12^	3.3 × 10^−11^	1.7 × 10^−9^
hsa-miR-122-3p	26.60	−2.67	−6.39	1.7 × 10^−12^	3.9 × 10^−11^	2.1 × 10^−9^
hsa-miR-30c-5p	65.96	−1.26	−2.40	5.6 × 10^−12^	1.3 × 10^−10^	7.1 × 10^−9^
hsa-miR-200b-3p	7.40	−2.88	−7.37	1.3 × 10^−11^	2.8 × 10^−10^	1.6 × 10^−8^
hsa-miR-365a-3p	10.24	−2.27	−4.84	1.6 × 10^−11^	3.4 × 10^−10^	2.1 × 10^−8^
hsa-miR-455-3p	6.60	−2.97	−7.84	2.7 × 10^−11^	5.4 × 10^−10^	3.4 × 10^−8^
hsa-miR-574-3p	65.44	−1.74	−3.35	4.0 × 10^−11^	7.7 × 10^−10^	5.0 × 10^−8^
hsa-miR-378a-5p	4.76	−2.08	−4.23	1.2 × 10^−8^	2.2 × 10^−7^	1.5 × 10^−6^
hsa-miR-223-3p	3152.68	1.63	3.09	1.3 × 10^−8^	2.3 × 10^−7^	1.6 × 10^−5^
hsa-miR-455-3p	2.84	−2.75	−6.73	2.4 × 10^−8^	4.2 × 10^−7^	3.0 × 10^−5^
hsa-miR-203a-3p	10.84	−1.85	−3.59	3.4 × 10^−8^	5.8 × 10^−7^	4.3 × 10^−5^
hsa-miR-142-5p	156.32	1.29	2.45	4.8 × 10^−8^	8.0 × 10^−7^	6.1 × 10^−5^
hsa-miR-30e-3p	39.96	−1.46	−2.75	8.1 × 10^−8^	1.3 × 10^−7^	1.0 × 10^−4^
hsa-miR-28-3p	140.84	−0.86	−1.81	8.6 × 10^−8^	1.3 × 10^−7^	1.1 × 10^−4^
hsa-miR-1468-5p	2.40	−2.33	−5.02	1.7 × 10^−7^	2.6 × 10^−7^	2.2 × 10^−4^
hsa-miR-378i	4.44	−1.85	−3.60	1.9 × 10^−7^	2.8 × 10^−7^	2.4 × 10^−4^
hsa-miR-625-3p	86.60	1.37	2.58	3.7 × 10^−7^	5.3 × 10^−7^	4.7 × 10^−4^
hsa-miR-3065-5p	4.52	−1.96	−3.90	3.8 × 10^−7^	5.4 × 10^−7^	4.8 × 10^−4^
hsa-miR-874-3p	10.28	−1.51	−2.84	5.6 × 10^−7^	7.7 × 10^−7^	7.0 × 10^−4^

**Table 3:** Comparison of miRNA profiles in ENI vs. CHD patients: a total of 34 miRNAs showed significant differential expression after Bonferroni correction (cut-off = 0.001).

Assay	Max Groupmean	log2 Foldchange	Foldchange	*p*-Value	FDR *p*-Value	Bonferroni
hsa-miR-885-3p	48.16	−3.37	−10.32	6.3 × 10^−25^	3.9 × 10^−22^	8.0 × 10^−22^
hsa-miR-885-5p	70.76	−2.71	−6.53	2.2 × 10^−19^	6.5 × 10^−17^	2.8 × 10^−16^
hsa-miR-193b-5p	46.92	−2.74	−6.68	3.2 × 10^−19^	6.5 × 10^−17^	4.0 × 10^−16^
hsa-miR-100-5p	116.84	−2.40	−5.28	5.2 × 10^−18^	7.6 × 10^−16^	6.5 × 10^−15^
hsa-miR-34a-5p	41.88	−2.65	−6.29	6.2 × 10^−18^	7.6 × 10^−16^	7.8 × 10^−15^
hsa-miR-192-5p	1675.12	−2.32	−5.00	1.8 × 10^−17^	1.9 × 10^−15^	2.3 × 10^−14^
hsa-miR-148a-3p	2186.08	−1.60	−3.02	2.3 × 10^−17^	2.0 × 10^−15^	2.8 × 10^−14^
hsa-miR-122-5p	140,835.00	−2.31	−4.96	1.4 × 10^−15^	1.0 × 10^−13^	1.7 × 10^−12^
hsa-miR-194-5p	600.92	−1.97	−3.93	1.6 × 10^−15^	1.1 × 10^−13^	2.0 × 10^−12^
hsa-miR-27b-3p	295.20	−1.47	−2.77	1.1 × 10^−14^	6.6 × 10^−13^	1.4 × 10^−11^
hsa-miR-365a-3p	21.28	−2.51	−5.70	1.7 × 10^−14^	9.4 × 10^−13^	2.1 × 10^−11^
hsa-miR-483-3p	253.28	−2.30	−4.93	3.1 × 10^−14^	1.6 × 10^−12^	3.9 × 10^−11^
hsa-miR-23b-3p	255.72	−1.96	−3.90	3.9 × 10^−14^	1.8 × 10^−12^	4.9 × 10^−11^
hsa-miR-125b-5p	2728.28	−1.84	−3.57	6.0 × 10^−14^	2.6 × 10^−12^	7.5 × 10^−11^
hsa-miR-30a-3p	45.40	−2.18	−4.54	6.3 × 10^−14^	2.6 × 10^−12^	8.0 × 10^−11^
hsa-miR-99a-5p	179.40	−1.93	−3.80	1.7 × 10^−13^	6.6 × 10^−12^	2.1 × 10^−10^
hsa-miR-146b-5p	175.20	−1.91	−3.75	4.3 × 10^−13^	1.6 × 10^−11^	5.5 × 10^−10^
hsa-miR-122-3p	54.72	−2.66	−6.33	1.3 × 10^−12^	4.4 × 10^−11^	1.6 × 10^−9^
hsa-miR-146b-3p	16.12	−2.54	−5.83	1.9 × 10^−12^	6.1 × 10^−11^	2.3 × 10^−9^
hsa-let-7c-5p	1429.64	−2.03	−4.09	2.3 × 10^−12^	7.1 × 10^−11^	2.9 × 10^−9^
hsa-miR-194-3p	8.08	−3.78	−13.76	4.8 × 10^−12^	1.4 × 10^−10^	6.1 × 10^−9^
hsa-miR-30c-5p	122.72	−1.24	−2.37	5.4 × 10^−12^	1.5 × 10^−10^	6.8 × 10^−9^
hsa-miR-574-3p	141.24	−1.80	−3.47	6.1 × 10^−12^	1.6 × 10^−10^	7.7 × 10^−9^
hsa-miR-125b-2-3p	6.44	−3.22	−9.29	2.3 × 10^−11^	5.9 × 10^−10^	2.9 × 10^−8^
hsa-miR-378a-5p	10.60	−2.33	−5.02	2.4 × 10^−11^	5.9 × 10^−10^	3.0 × 10^−8^
hsa-miR-30a-5p	335.88	−1.29	−2.45	2.9 × 10^−11^	7.0 × 10^−10^	3.7 × 10^−8^
hsa-miR-455-5p	10.16	−2.66	−6.31	1.4 × 10^−9^	3.2 × 10^−8^	1.8 × 10^−6^
hsa-miR-1468-5p	4.40	−2.44	−5.44	8.6 × 10^−9^	1.9 × 10^−7^	1.1 × 10^−5^
hsa-miR-193a-5p	133.12	−1.04	−2.06	1.3 × 10^−8^	2.7 × 10^−7^	1.6 × 10^−5^
hsa-miR-204-5p	12.04	−1.68	−3.20	6.8 × 10^−8^	1.4 × 10^−6^	8.6 × 10^−5^
hsa-miR-345-5p	25.48	−1.24	−2.36	8.5 × 10^−8^	1.7 × 10^−6^	1.1 × 10^−4^
hsa-miR-28-3p	244.56	−0.81	−1.75	3.3 × 10^−7^	6.4 × 10^−6^	4.1 × 10^−4^
hsa-miR-99a-3p	4.16	−2.49	−5.60	5.4 × 10^−7^	1.0 × 10^−5^	6.8 × 10^−4^
hsa-miR-455-3p	4.08	−2.40	−5.28	6.3 × 10^−7^	1.2 × 10^−5^	8.0 × 10^−4^

**Table 4:** miRNAs with significant differential expression between ENI and CHD but not between ENI and CHB.

	ENI vs. CHD		ENI vs. CHB	
Assay	Fold Change	Bonferroni	Fold Change	Bonferroni
hsa-miR-204-5p	−3.20	8.60 × 10^−05^	−2.89	3.16 × 10^−03^
hsa-miR-345-5p	−2.36	1.08 × 10^−04^	−2.21	2.84 × 10^−03^
hsa-miR-99a-3p	−5.60	6.75 × 10^−04^	−4.89	1.37 × 10^−02^

**Table 5:** miRNAs with significant differential expressions between ENI and CHB but not between ENI and CHD.

	ENI vs. CHB		ENI vs. CHD	
Assay	Fold Change	Bonferroni	Fold Change	Bonferroni
hsa-miR-378a-3p	−2.78	1.32 × 10^−10^	−1.90	3.18 × 10^−03^
hsa-miR-200a-3p	−7.05	1.56 × 10^−09^	−2.04	1.00
hsa-miR-200b-3p	−7.37	1.64 × 10^−08^	−2.90	4.34 × 10^−01^
hsa-miR-203a-3p	−3.59	4.31 × 10^−05^	−3.02	1.44 × 10^−03^
hsa-miR-30e-3p	−2.75	1.03 × 10^−04^	−2.46	1.67 × 10^−03^
hsa-miR-378i	−3.60	2.37 × 10^−04^	−3.00	4.52 × 10^−03^
hsa-miR-3065-5p	−3.90	4.82 × 10^−04^	−2.40	1.00
hsa-miR-874-3p	−2.84	7.03 × 10^−04^	−2.19	1.39 × 10^−01^

## Data Availability

The data presented in this study are available on request from the corresponding author.

## Supplementary Materials

The following supporting information can be downloaded at: https://www.mdpi.com/article/10.3390/v15112257/s1, Table S1: miRNAs with significant differential expression between ENI vs. CHB, but not between ENI and CHD.
