# Supplementary material for: The Circulating miRNA Profile of Chronic Hepatitis D and B Patients Is Comparable but Differs from That of Individuals with HBeAg-Negative HBV Infection
Source: Viruses. 2023 Nov 15;15(11):2257. doi: 10.3390/v15112257 (PMC10675264; doi:10.3390/v15112257)
Supplement: Supplementary file 1 [file viruses-15-02257-s001.zip › viruses-2693620-supplementary.pdf]

Supplementary Table S1: miRNAs with significant differential expression between ENI vs. CHB, but not between ENI and CHD.

	ENI vs. CHB		ENI vs. CHD	
**Assay**	**Fold change**	**Bonferroni**	**Fold change**	**Bonferroni**
hsa-miR-625-3p	2.58	$4.7x10^{-4}$	2.50	$8.01x10^{-1}$
hsa-miR-142-5p	2.45	$6.1x10^{-5}$	1.79	$3.82x10^{-1}$
hsa-miR-223-3p	3.09	$1.6x10^{-5}$	1.63	1.00
